# Efficient 2G Ethanol Production via Optimized Dilute Acid Pretreatment, High-Solids Enzymatic Hydrolysis, and High-Temperature Fermentation

**DOI:** 10.1021/acsomega.5c11192

**Published:** 2026-01-08

**Authors:** Isabela S. Ferreira, Miguel A. D. Flores-Alarcón, Inês C. Roberto

**Affiliations:** Department of Biotechnology, 28133 Engineering College of Lorena, University of São Paulo (USP), Estrada Municipal do Campinho, N° 100, Campinho, Lorena, SP 12602-810, Brazil

## Abstract

The growing demand for 2G ethanol emphasizes the need to improve its economic sustainability through different approaches, including optimized pretreatments, high-solids enzymatic hydrolysis, and high-temperature fermentation. This study evaluated the dilute acid pretreatment of deacetylated rice straw using the response surface methodology. Under optimal conditions (85 mg of H_2_SO_4_/g biomass, for 10 min at a constant temperature of 150 °C), the process was scaled up to an 80 L reactor, obtaining a cellulose-rich (58.2% w/w) pretreated solid. Then, high-solids enzymatic hydrolysis at 24% w/v solids loading was performed in a vertical ball mill (VBM) reactor in fed-batch mode, resulting in a hydrolysate of 129 g/L of fermentable sugars, which corresponded to a cellulose conversion yield (CCY) of 78.6%. Finally, the effect of nutritional supplementation of the obtained slurry and hydrolysate on the fermentability of *Kluyveromyces marxianus* at 43 °C was studied in conical flasks under orbital shaking. The higher $Y_{P/S}$ (0.46 g/g), *Q*_P_ (1.74 g/L/h), and η (90%) were achieved in the hydrolysate + nutrients medium. Additionally, the effect of the VBM reactor on ethanol production was evaluated, further increasing *Q*_P_ (3.04 g/L/h) with an ethanol titer of 37 g/L. Therefore, the processing sequential steps and conditions efficiently produced a glucose-rich hydrolysate, which was successfully fermented into ethanol at high temperatures and could support the process feasibility at a large scale.

## Graphical abstract

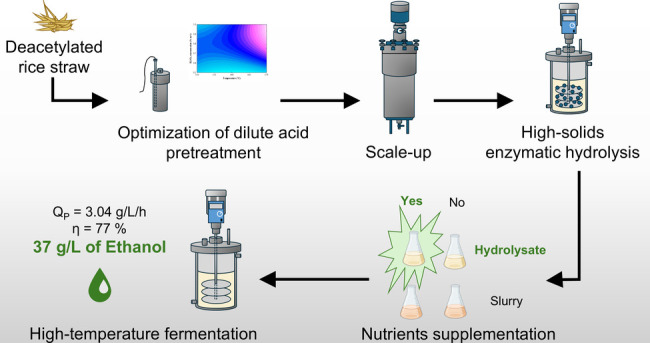

## 1 Introduction

Ethanol is nowadays the most widely used renewable fuel for transportation, with its global production increasing to satisfy growing energy demands and reduce future dependence on fossil fuels.^1^ In 2024, production of this biofuel was estimated to be around 16.2 million gallons worldwide, with the United States (52%) and Brazil (28%) being the largest producers.^2^ Industrially, first-generation (1G) ethanol production (from food-based feedstocks such as corn and sugar cane) is a well-established process.^3^ In Brazil, there are currently second-generation (2G) ethanol plants, GranBio and Raízen—which implemented standalone (just 2G ethanol production) and integrated (1G+2G ethanol production) process configurations, respectively. These two 2G ethanol plants have achieved commercial-scale production due to their business models.^4^ However, there are still challenges that hinder economic sustainability of 2G ethanol plants, including high cost of chemicals for pretreatment, low solids content used in the enzymatic hydrolysis step, and incomplete utilization of all major components (cellulose, hemicellulose, and lignin) of lignocellulosic biomass.^5^

Since lignocellulosic biomass (byproducts of agricultural, industrial, and forestry activities) is used as a raw material for producing 2G ethanol, it is possible to obtain other value-added products from lignocellulosic biorefinery, offering a sustainable development.^6^ One of the potential raw materials for producing 2G ethanol is rice straw, due to its large availability as a byproduct of rice harvesting and its high cellulose and hemicellulose content, which can be hydrolyzed into fermentable sugars.^7,8^ For this purpose, various integrated processes are available, including separate hydrolysis and fermentation (SHF) and simultaneous saccharification and fermentation (SSF).^9^ As for the temperature for 2G ethanol fermentation, it is known that 30–35 °C is the ideal range for the frequently studied *Saccharomyces cerevisiae*.^10^ However, thermotolerant ethanol producing yeasts (35–45 °C), such as *Kluyveromyces marxianus*, have demonstrated to be a promising alternative, achieving high volumetric productivity and ethanol titers above 50 g/L.^11,12^ In addition, 2G ethanol production at high temperatures has been assessed as more economical, practical, and advantageous for large-scale production.^13,14^

Regarding enzymatic hydrolysis, it corresponds to a surface phenomenon (cellulases directly contacting the substrate),^15^ occurring under mild conditions of pH (4.8) and temperature (45 to 50 °C).^16^ The obtention of a hydrolysate with a high sugar concentration is crucial for making the fermentation step technically and economically viable, with one approach being the use of high solids loadings (>15% w/w).^17^ However, in addition to the major barrier, which is its natural recalcitrance, lignocellulosic feedstock poses several challenges for high-solids enzymatic hydrolysis including mixing, mass transfer, product inhibition, and higher cellulase adsorption.^18^

Primarily, to overcome the recalcitrance of lignocellulosic biomass for 2G ethanol production, pretreatment is the essential technical step.^19^ In this regard, Castro et al.^20^ defined a strategy consisting of a deacetylation prior to dilute acid pretreatment of rice straw, improving the recovery of cellulose and hemicellulose fractions and, subsequently, the SSF process. However, a techno-economic assessment^21^ has revealed that the dilute acid pretreatment is one of the most expensive processing steps of rice straw biorefineries for producing 2G ethanol, hence the need to adjust its conditions for reduced energy demands (temperature and time).

About overcoming the challenges caused by high-solids enzymatic hydrolysis, a feeding strategy consisting of gradually adding the substrate has been described as a promising alternative for industrial-scale application.^12^ Along with it, the use of nonconventional reactors can improve the hydrolysis performance by promoting adequate mixing and thereby reducing mass and heat transfer limitations.^22,23^ The nonconventional reactors, which differ from the standard stirred-tank configuration by presenting, for example, alterations in the impeller-type, can also be used in fermentation processes for producing ethanol.^22,24^ In our previous work,^12^ a vertical ball mill reactor (VBM) was used for performing an SSF process at high solid loadings of pretreated rice straw with gradual feeding of substrate and employing a thermotolerant yeast strain (*K. marxianus* NRRL Y-6860). Although the overall process (hydrolysis and fermentation) efficiency was high (67%), the SSF process was limited due to the incomplete hydrolysis of cellulose.

In this context, the present work aims to optimize the dilute acid pretreatment of deacetylated rice straw and then to evaluate the 2G ethanol production by the SHF process in a nonconventional reactor. The high-solids enzymatic hydrolysis was performed with a gradual feeding strategy (16 + 4 + 4% solids loadings). Subsequently, the fermentability of the obtained hydrolysate (liquid fraction) and slurry (hydrolysate + residual solids) was evaluated employing *K. marxianus* yeast, and the effect of nutrient supplementation on ethanol production was studied, in shake flasks. Additionally, the fermentation step was conducted under more favorable conditions in the VBM reactor. This is the first time that the VBM reactor is used for the SHF process.

## 2 Materials and Methods

### 2.1 Obtention of Deacetylated Rice Straw

Rice straw collected from fields in the Canas/SP region of Brazil was used as the feedstock. Initially, the lignocellulosic material was naturally dried until it reached about 10% moisture content and then hammer-milled to obtain particles measuring approximately 1 × 1 cm (length × thickness). This material, namely, raw rice straw, was stored at room temperature for further processing.

The deacetylation process was carried out in a 50 L batch reactor under previously defined conditions (80 mg of NaOH/g of biomass, 70 °C, 45 min, solid-to-liquid ratio of 1:10).^20^ The composition of the raw and deacetylated rice straw was determined according to the NREL-LAP standard protocol.^25^

### 2.2 Evaluation of Dilute Acid Pretreatment Using Experimental Design

The dilute acid pretreatment of deacetylated rice straw was evaluated using different conditions of temperature (150–170 °C) and concentrations of H_2_SO_4_ solution (0.5–1.0% w/v) combined according to a 2^2^ face-centered central composite design (FC-CCD). For all these experiments, 20 g (dry mass) of deacetylate rice straw was placed in 0.5 L stainless-steel reactors and impregnated with the corresponding H_2_SO_4_ solution at a solid-to-liquid ratio of 1:10. Then, the reactors were heated in a silicone oil bath controlled with an immersion thermostat (E200, Lauda). Since the temperature of the silicone oil bath differs from the internal temperature of the reactor, the latter was measured using a thermocouple and considered as the reaction temperature. For each assay of FC-CCD, 20 min at a constant temperature was resolved. Additionally, the combined severity factor (CSF) of each assay was calculated using eq 1, for including the effect of time, temperature, and acid concentration into a single variable as described by Guo et al.^26^ and Lloyd and Wyman,^27^ which in turn were based on the work of Chum et al.^28^

$$\mathrm{CSF}=\log[t\times e^{\left(T_{H}-T_{R}/14.75\right)}]-\mathrm{pH}$$ 1

where *t* is the hydrolysis time (min), *T*_H_ is the hydrolysis temperature (°C), *T*_R_ is the reference temperature (100 °C), and pH is the acidity function indicator.

After the reaction, the pretreated solids (cellulosic-lignin residue) were separated from the hemicellulosic (acid) hydrolysate by filtration using 120 mesh sieves. These solids were then washed with water until neutral pH (∼6.5) and dried naturally to a moisture content of about 10%. Lastly, it was weighed and chemically characterized (NREL-LAP standard protocol),^25^ to calculate the % of mass recovery (MR) and cellulose recovery (CR). The hemicellulosic hydrolysate was also analyzed for determining monomeric sugars (glucose, xylose, and arabinose) and byproduct (furans and phenolic compounds) concentrations. Based on the quantification of solubilized sugars, the hemicellulose hydrolysis efficiency (HHE) was calculated as detailed elsewhere.^20^

### 2.3 Dilute Acid Pretreatment Kinetics and Scale-up

Under optimized conditions (85 mg of H_2_SO_4_/g of biomass, at 150 °C), the influence of total time (ramp time + time at constant temperature), from the moment the reactor is immersed in the silicone oil bath, was evaluated. This way, the monitoring time involved a ramp time (heating phase) that lasted 55 min, followed by 30 min at a constant temperature (150 °C), totaling a time of 85 min. These experiments were conducted in the 0.5 L stainless-steel reactor equipped with the thermocouple. After reactions, the pretreated solids and the hemicellulosic hydrolysate were separated and both analyzed, as detailed in Section 2.2.

To obtain an enough amount of pretreated solids for subsequent experiments of high-solids enzymatic hydrolysis, the dilute acid pretreatment under the optimal evaluated conditions (85 mg of H_2_SO_4_/g biomass, for 10 min at a constant temperature of 150 °C) was scaled up to an 80 L stainless steel reactor, using 3.2 kg (dry mass) of deacetylated rice straw with 32 L of H_2_SO_4_ solution. The obtained pretreated solids were washed with water until reaching a neutral pH, naturally dried (10% moisture content), and chemically characterized.^25^

### 2.4 High-Solids Enzymatic Hydrolysis in a Vertical Ball Mill (VBM) Reactor

The high-solids enzymatic hydrolysis was carried out in triplicate at 46 °C and 100 rpm, using a 1.5 L vertical ball mill (VBM) reactor (120 mm inner diameter) equipped with a three-flat-disk impeller (94 mm diameter), which were positioned at a distance of 28 mm each other. Above each flat disk, 10 glass spheres (23 mm diameter and 8.16 ± 0.35 g each) were placed as grinding elements.^22^ The hydrolysis process was carried out using a fed-batch strategy (16 + 4 + 4% w/v of pretreated solids), starting with 16% (w/v) of pretreated solids, and after 10 and 24 h, the reactor was fed with an additional 4% (w/v) of pretreated solids, as previously defined.^12^ The total enzyme loading used was 29.5 FPU/g of cellulose, which corresponded to a mix of Cellubrix (21.5 FPU/g of cellulose) + Novozyme 188 (26.5 IU/g of cellulose), both from Novozymes Corporation (Denmark). The resulting slurry (hydrolysate + residual solids) and hydrolysate (liquid fraction obtained by centrifugation at 1100 rpm for 20 min) were subsequently used for fermentation experiments. Additionally, hydrolysis experiments at solids loadings of 16, 20, and 24% w/v were carried out in batch mode as controls, under the abovementioned operational conditions.

During hydrolysis, samples were collected periodically, until 48 h, and immediately centrifuged at 4000 rpm × 10 min (Heraeus Megafuge 16R, Thermo Scientific). After centrifugation, the enzyme was inactivated by boiling for 5 min, and the supernatant was stored at 4 °C for further analysis of sugar concentrations (glucose, cellobiose, xylose, and arabinose). Based on the analyzed sugar concentrations, it was possible to calculate the cellulose conversion yield (CCY) as a percentage of the maximum theoretical conversion into glucose, using eq 2.

$$\mathrm{CCY}(\%)=\frac{\mathrm{glu}+(1.053\times \mathrm{ceb})}{C\times 1.11\times S}\times 100$$ 2

where glu and ceb are the glucose and cellobiose concentrations in the hydrolysate, respectively (g/L); *C* is the cellulose content in the pretreated solid (g/g); 1.053 and 1.11 correspond to the conversion factor of cellobiose (360/342) and cellulose (180/162) to glucose, respectively; and *S* is the pretreated solids content (g/L).

### 2.5 Microorganism and Inoculum

For high-temperature fermentation, the thermotolerant yeast strain *Kluyveromyces marxianus* NRRL Y-6860, which was maintained in malt extract agar at 4 °C until use, was employed. The inoculum preparation was carried out as described in previous work.^22^

### 2.6 Fermentability of Cellulosic Slurry and Hydrolysate with and without Nutrient Supplementation

For these experiments, the slurry and hydrolysate obtained by the high-solids enzymatic hydrolysis in batch mode at 24% w/v solids loadings was used. The evaluation of the fermentability of the hydrolysate and slurry as well as the effect of nutrient supplementation on ethanol production by *K. marxianus* was carried out in 125 mL Erlenmeyer flasks, containing 50 mL of fermentation medium: (i) just slurry, (ii) slurry + nutrients, (iii) just hydrolysate, and (iv) hydrolysate + nutrients.

The volume of fermentation medium consisted of 49 mL of hydrolysate/slurry (adjusted to a pH of 5.5 by adding NaOH 10 M) with 1 mL of nutrient solution. This way, the fermentation medium was composed of (g/L) glucose (∼78.0), xylose (∼12.0), and cellobiose (∼6.0). In the experiments with nutrient supplementation, sterilized concentrated solutions were added to obtain a fermentation medium with nutrient concentrations (g/L) of 1.0 for (NH_4_)_2_SO_4_, 1.5 for KH_2_PO_4_, 0.1 for MgSO_4_·7H_2_O, and 3.0 for yeast extract, while in the experiments without nutrients, these solutions were replaced by distilled sterile water at the same volume. The flasks containing the fermentation medium were inoculated with 3 g/L of initial cell concentration and incubated in a shaker (TE-420, Tecnal) at 43 °C and 100 rpm for 24 h. Periodic samples were withdrawn and centrifuged (4000 rpm × 10 min) to monitor sugar consumption and ethanol production.

### 2.7 Ethanol Production in the VBM Reactor

After the most appropriate fermentation medium (hydrolysate + nutrients) for ethanol production by *K. marxianus* was determined, the effect of the VBM reactor was evaluated. For this purpose, the VBM reactor was used in the absence of glass spheres, at 40 °C and 200 rpm.^22^ In the present study, the fermentation medium consisted of 0.49 L of the hydrolysate (obtained by the high-solids enzymatic hydrolysis in fed-batch mode and adjusted to a pH of 5.5 by adding NaOH 10 M) with 0.01 L of nutrient solutions, reaching a total final volume of 0.50 L. This way, fermentation medium was composed of (g/L) glucose (107.0), xylose (9.0), and cellobiose (12.0) from hydrolysate, supplemented with (NH_4_)_2_SO_4_ (1.0), KH_2_PO_4_ (1.5), MgSO_4_·7H_2_O (0.1), and yeast extract (3.0). The medium was inoculated with 3 g/L of initial cell concentration.

For comparison purposes, fermentation in the VBM reactor was carried out with a semi-defined medium with a composition similar to the hydrolysate + nutrient medium as a control, under the same abovementioned process conditions. Samples were periodically taken, until 12 h, to measure cell growth, substrate (glucose), and product (ethanol, glycerol, and acetic acid) concentrations.

### 2.8 Parameter Calculations for Fermentation

Fermentation profiles were analyzed using Origin software version 2024 (OriginLab Corporation, Northampton, Massachusetts, USA), fitting the kinetics (*R* ^2^ ≥ 0.8). Using the kinetic profiles, the ethanol yield from glucose ( $Y_{P/S}$ ), the ethanol efficiency (η), and the ethanol volumetric productivity (*Q*_P_) were calculated as specified elsewhere.^23^ *Q*_P_ was calculated at the time of maximum ethanol concentration.

### 2.9 Analytical Methods

Cell (*K. marxianus*) growth was determined by measuring the optical density (OD) at 600 nm in a UV–vis spectrophotometer (U-1800, Hitachi). The OD measured was converted to cell concentration using a suitable calibration curve (cells (g/L) = 3.89 × OD + 0.02), correlating OD × dry weight.

The concentrations of glucose, xylose, arabinose, acetic acid, glycerol, and ethanol were determined by high-performance liquid chromatography (HPLC) (Agilent Technologies 1260 Infinity), equipped with a refractive index detector (RID) and a Bio-Rad Aminex HPX-87H column (300 × 7.8 mm), at conditions detailed by Silva et al.^23^

The concentrations of furans (furfural, 5-hydroximethylfurfural (5-HMF), and furoic acid) and low molecular weight phenolics (gallic, vanillic, *p*-coumaric, syringic, and ferulic acids; vanillyl alcohol; and vanillin) were also determined by HPLC but equipped with a UV detector (at 276 nm) and a Waters Spherisorb S5ODS2 C18 5 μm (4.6 mm × 100 mm) column, at conditions described elsewhere.^29^

### 2.10 Statistical Analysis

The experimental obtained data was analyzed using Statistica v14.0 (TIBCO Software Inc., 2020, San Ramon, California, USA) and Design-Expert software v12.0 (Stat-Ease, Inc., Minneapolis, Minnesota, USA), considering a 95% confidence level. The importance and magnitude of the effects on the independent variables were determined by Pareto charts. Analysis of variance (ANOVA) was performed to determine the significance of the models and coefficients (*R* ^2^). Then, the optimized levels were obtained by the numerical optimization function of the software.

## 3 Results and Discussion

### 3.1 Optimization of Dilute Acid Pretreatment by the Response Surface Method

The effects of the different conditions of dilute acid pretreatment on the chemical composition of obtained pretreated solids and hemicellulosic hydrolysate (Table S1), according to the FC-CCD, were expressed in terms of cellulose recovery (CR), mass recovery (MR), hemicellulose hydrolysis efficiency (HHE), and concentrations of furans (*C*_Fur_) and phenolics (*C*_Phe_) in the hemicellulosic hydrolysate, as summarized in Table 1.

**Table 1:** Effects of Different Conditions of Dilute Acid Pretreatment According to a Face-Centered Central Composite Design (FC-CCD)

	independent variables^b^			responses^d^				
assay^a^	*X*_1_	*X*_2_	CSF^c^	CR	MR	HHE	*C*_Fur_	*C*_Phe_
1	150	0.50	1.44	87.49	60.78	69.94	0.20	1.17
2	170	0.50	2.03	90.25	59.91	63.24	0.48	6.11
2 (Rep)	170	0.50	2.03	90.59	59.38	61.20	0.47	3.67
3	150	1.00	1.67	87.93	57.78	80.14	0.27	2.60
3 (Rep)	150	1.00	1.67	86.86	55.76	82.53	0.31	1.01
4	170	1.00	2.26	56.63	34.35	44.30	0.42	5.09
5	150	0.75	1.60	81.94	54.36	80.03	0.20	1.99
6	170	0.75	2.19	71.23	45.79	56.94	0.37	5.08
7	160	0.50	1.74	89.76	60.10	73.20	0.26	1.72
8	160	1.00	1.97	88.70	57.83	77.87	0.31	2.58
9 (Cp)	160	0.75	1.90	75.95	47.48	70.14	0.39	3.45
10 (Cp)	160	0.75	1.90	81.10	51.78	76.70	0.32	1.91

a Rep = replicate, Cp = central point.

b *X*_1_ = temperature (°C), *X*_2_ = H_2_SO_4_ concentration (% w/v).

c CSF = combined severity factor.

d CR = cellulose recovery (%), MR = mass recovery (%), HHE = hemicellulose hydrolysis efficiency (%), *C*_Fur_ = concentration of furans (g/L), and *C*_Phe_ = concentration of phenolics in the hemicellulosic hydrolysate (g/L).

As can be noted (Table 1), CR values varied widely from 56.6 (assay 4) to 90.6% (assay 2), with the lower values observed under conditions involving high H_2_SO_4_ concentrations and temperature (assays 4 and 6). This behavior can be principally attributed to the susceptibility of the amorphous regions of cellulose to acid hydrolysis under severe conditions.^30^ A similar trend was noted for the MR values due to cellulose and hemicellulose removal. Concerning HHE, this value varied from 44.3 (assay 4) to 82.5% (assay 3), showing improvement under lower temperature conditions and higher H_2_SO_4_ concentrations. Thus, an increase in temperature negatively affected the HHE, under conditions corresponding to assays 2, 4, and 6. In these same assays, the higher concentrations of furfural, 5-HMF, and furoic acid were revealed, with the highest *C*_Fur_ reaching 0.48 g/L (assay 2). These results indicate that the highest evaluated temperature (170 °C) intensified hemicellulose degradation into furans during acid pretreatment. Additionally, *C*_Phe_ values of around 5.4 g/L were detected under more severe pretreatment conditions (assays 2, 4, and 6), due to the partial lignin disruption.^31^

In the design of experiments (Table 1), the calculation of CSF was useful to combine the temperature and H_2_SO_4_ concentrations in a meaningful way, also considering the effect of time. However, the different evaluated responses may not necessarily follow a trend, limiting the accuracy of performance predictions with the sole use of the CSF.^27^ Therefore, a statistical analysis was conducted for better determination of significant individual and interaction effects between the two factors (temperature and H_2_SO_4_ concentration) on the response variables (CR, MR, HHE, *C*_Fur_, and *C*_Phe_). Prior to performing the analysis of variance (ANOVA), the assumptions of normality and homogeneity of variances were verified. As a result, the Pareto charts are shown in Figure 1, revealing the significant (*p*-value <0.05) effect of pretreatment conditions on the evaluated responses.

**Figure 1:**
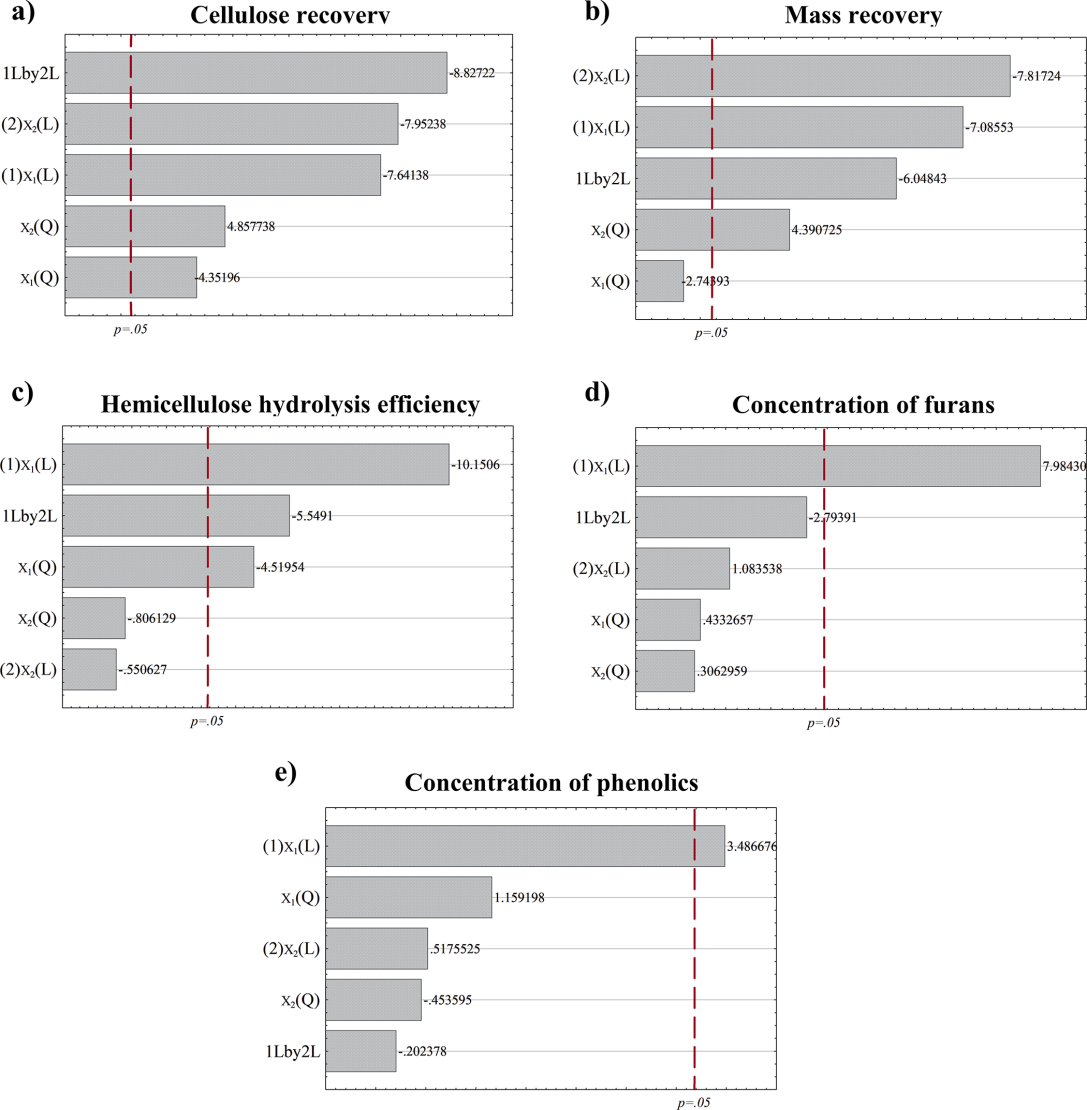
Pareto charts of the standardized effects of temperature (*X*_1_) and H_2_SO_4_ concentration (*X*_2_) on response variables: (a) cellulose recovery (CR), (b) mass recovery (MR), (c) hemicellulose hydrolysis efficiency (HHE), (d) concentration of furans (*C*_Fur_), and (e) concentration of phenolics (*C*_Phe_).

With regard to the effects on CR (Figure 1a), all of these were statistically significant, including the main effects of temperature (*X*_1_) and H_2_SO_4_ concentration (*X*_2_), interaction (*X*_1_·*X*_2_), and both quadratic effects (*X*_1_^2^ and *X*_2_^2^). Since *X*_1_^2^ and *X*_2_^2^ terms were significant with negative (−4.4) and positive (+4.8) effects, respectively, there was an expressed curvature within the model for CR. This behavior proves that CR increased as temperature and H_2_SO_4_ concentration increased at a certain point and then decreased due to severity. MR presented the same behavior with all of the statistically significant effects, excluding *X*_1_^2^ (Figure 1b). As with CR, MR can be adjusted to a second-order model. Similar impacts of temperature and H_2_SO_4_ on cellulose and lignin content were determined in the study of Martins et al.^30^

Concerning the Pareto chart for HHE (Figure 1c), the *X*_1_ term showed the largest and most significant negative effect (−10.2), indicating that lower temperatures improved the solubilization of xylose and arabinose in the hemicellulosic hydrolysate. It is also worth noting that the *X*_1_·*X*_2_ effect was significant, which revealed the dependence of the H_2_SO_4_ concentration on the temperature level to improve HHE. This variable significance should be interpreted with caution, considering that the H_2_SO_4_ concentration was evaluated in a short range (0.5–1.0%), with the highest level producing the highest HHE. Additionally, for both *C*_Fur_ (Figure 1d) and *C*_Phe_ (Figure 1e), it was found that *X*_1_ presented the only statistically significant and positive effect. This effect suggests that the higher *C*_Fur_ and *C*_Phe_ resulting from hemicellulose and lignin degradation, respectively, are directly associated with high temperatures during dilute acid pretreatment.

For all response variables (CR, MR, HHE, *C*_Fur_, and *C*_Phe_), non-significant terms (*p*-values >0.05) were removed aiming to reduce the model and increase fitting (*R* ^2^). Thus, adjusted mathematical models describing the behavior of each response within the evaluated range of temperature and H_2_SO_4_ concentration were obtained in coded values (eqs 3, 4, 5, 6, and 7). Response surface models were also constructed from these equations (Figure S1). In general, the different behaviors of the variables highlighted the importance of optimizing the dilute acid pretreatment through an experimental design to enhance the process performance.

$$\mathrm{CR}(\%)=80.54-6.00X_{1}-6.25X_{2}-8.17X_{1}X_{2}-5.98X_{1}^{2}+6.67X_{2}^{2}\;(R^{2}=0.88)$$ 3

$$\mathrm{MR}(\%)=52.89-5.05X_{1}-5.57X_{2}-5.32X_{1}X_{2}\;(R^{2}=0.74)$$ 4

$$\mathrm{HHE}(\%)=74.48-11.02X_{1}-0.5977X_{2}-6.96X_{1}X_{2}-8.92X_{1}^{2}\;(R^{2}=0.95)$$ 5

$$C_{\mathrm{Fur}}(g/L)=0.3257+0.0999X_{1}+0.0136X_{2}-0.0431X_{1}X_{2}\;(R^{2}=0.86)$$ 6

$$C_{\mathrm{Phe}}(g/L)=3.03+1.71X_{1}+0.2538X_{2}\;(R^{2}=0.73)$$ 7

Although the present study focused on obtaining a pretreated solid with high cellulose content for high-solids enzymatic hydrolysis and subsequent fermentation, obtaining a hemicellulosic-rich hydrolysate with low levels of toxic compounds (furans and phenolics) is also essential to avoid a detoxification step prior to its use in biochemical processes, such as fermentation.^31,32^ Therefore, a multicriteria optimization was considered using the numerical optimization function of Design-Expert 12.0 software, and the desirability response surface can be seen in Figure S2. This approach combined multiple response variables to identify a single optimized condition by maximizing the CR, MR, and HHE (%) while minimizing *C*_Fur_ and *C*_Phe_ (g/L).

The optimized levels were defined at a temperature of 150 °C and 0.85% w/v of H_2_SO_4_ concentration. Under these conditions, the predicted responses were CR of 82.41 ± 4.73%, MR of 57.84 ± 4.74%, HHE of 79.12 ± 3.34%, *C*_Fur_ of 0.25 ± 0.04 g/L, and *C*_Phe_ of 1.42 ± 0.96 g/L. Thus, validation experiments were conducted, resulting in CR of 93.0 ± 5.7%, MR of 62.6 ± 4.3%, HHE of 78.7 ± 3.2%, *C*_Fur_ of 0.263 ± 0.030 g/L, and *C*_Phe_ of 1.119 ± 0.056 g/L. These results demonstrated a good correlation between experimental and predicted values, indicating that the adjusted mathematical models can be used to predict each studied response within the evaluated range of temperature and H_2_SO_4_ concentration. Furthermore, obtaining a cellulose-rich solid along with a hemicellulose-rich hydrolysate with low levels of inhibitors (phenolics and furans) is advantageous for the bioconversion of both streams to 2G ethanol. Alternatively, the conversion of hemicellulose hydrolysate into high-value compounds, such as xylitol, has proven to be more profitable in a biorefinery scenario.^21^

### 3.2 Effect of Time on Dilute Acid Pretreatment under Optimized Conditions

Considering that the time of dilute acid pretreatment in the FC-CCD was fixed at 20 min at a constant temperature, additional experiments were conducted under optimized conditions (150 °C and 0.85% w/v of H_2_SO_4_ concentration) for monitoring the effect of total time (ramp time + time at a constant temperature) on the chemical composition of pretreated solid and the hemicellulosic hydrolysate. As a result, the CR (%), MR, and HHE (%) were calculated, and their kinetic profiles over time are presented in Figure 2.

**Figure 2:**
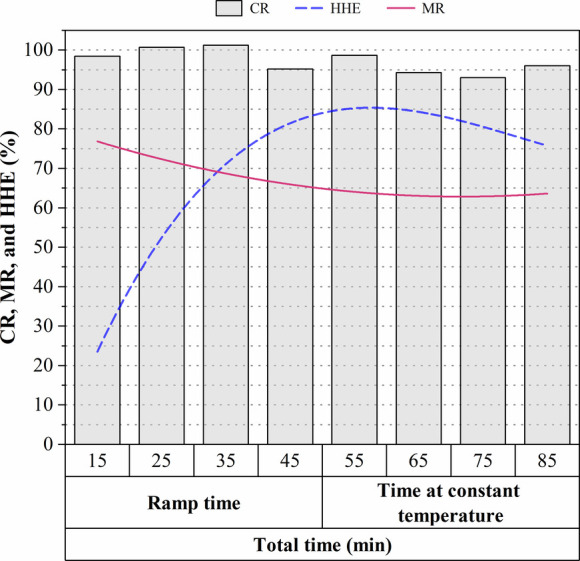
Effect of time on cellulose recovery (CR), mass recovery (MR), and hemicellulose hydrolysis efficiency (HHE), during dilute acid pretreatment at a constant temperature of 150 °C using 84 mg of H_2_SO_4_ /g biomass, in 0.5 L stainless-steel reactors.

As can be seen in Figure 2, MR showed minimal variation between 35 and 85 min of residence time, ranging from 68 to 63%, approximately. Thus, the greatest loss of mass was observed in the first 25 min (ramp time). As the CR values were above 90% throughout the monitored time (85 min), it can be indicated that most of the mass loss corresponded to the hemicellulose fraction, which is susceptible to acid hydrolysis. The kinetic profile of HHE, in fact, showed a rapid and progressive increase, reaching a maximum value of 85% after a total time of 55 min, which corresponds to 10 min at a constant temperature of 150 °C. At this point, the highest values of xylose (19.1 g/L) and arabinose (4.3 g/L) were also detected. However, the xylose concentration decreased slightly after 65 min (data not shown), negatively affecting the HHE. The hemicellulosic hydrolysate was also analyzed for by product concentrations, with the highest levels detected at longer times. *C*_Fur_ was less than 0.33 g/L, while a maximum peak of 1.26 g/L *C*_Phe_ was detected after the total residence time. This behavior indicates that C5 sugars from arabinoxylan chain,^7^ once solubilized in the acid hydrolysate, began to degrade into furans (such as furfural and furoic acid) by oxidation, with the increase in time. It should also be mentioned that hemicellulose fraction can be more vulnerable to degradation due to the modification of the biomass structure and composition (lignin removal) by the deacetylation step,^20^ also explaining the low concentration (≤0.35 g/L) of acetic acid detected in the hemicellulosic hydrolysate. Consequently, the obtained concentrations of inhibitors were lower than that observed in other studies,^31,33^ suggesting that the hemicellulosic hydrolysate obtained in this study can be used for fermentation without additional detoxification.

Based on the obtained CR, MR, HHE, *C*_Fur_, and *C*_Phe_ over time, an optimal time of 10 min at constant temperature (150 °C) was established for the dilute acid pretreatment (85 mg of H_2_SO_4_/g of biomass), corresponding to a CSF of 1.25. It is important to note that the duration of the acid treatment was reduced by 10 min compared to that initially used in the FC-CCD. This reduction in time could benefit the profitability of the 2G ethanol production process from rice straw, considering that dilute acid pretreatment has been assessed as the most expensive step.^21^

Under the optimal conditions (85 mg of H_2_SO_4_/g of biomass, for 10 min at constant temperature of 150 °C), the dilute acid pretreatment was conducted on a larger scale (80 L reactor). As a result, the chemical compositions of the pretreated solids and the mass balance of the process are presented in Table 2.

**Table 2:** Chemical Composition of Raw Rice Straw, Deacetylated, and Pretreated Solids

	composition (g/100 g)				
components	raw rice straw	deacetylated	pretreated solids^a^	recovery^b^ (%)	recovery^c^ (%)
glucan	34.56 ± 0.5	42.07 ± 1.3	58.24 ± 1.4	82.4	69.2
hemicellulose	22.01 ± 0.3	23.95 ± 1.0	5.89 ± 0.2	14.6	11.0
xylan	19.29 ± 0.2	20.52 ± 0.9	5.89 ± 0.2	17.1	12.5
arabinan	2.72 ± 0.1	3.44 ± 0.2	not detected	0.0	0.0
acetyl groups	2.50 ± 0.5	0.41 ± 0.0	not detected	0.0	0.0
lignin	15.50 ± 0.9	12.47 ± 0.8	19.37 ± 0.2	92.5	51.3
acid insoluble lignin (AIL)	12.90 ± 0.8	10.94 ± 0.8	18.28 ± 0.3	99.5	58.2
acid soluble lignin (ASL)	2.60 ± 0.1	1.53 ± 0.2	1.09 ± 0.1	42.4	17.2
ash	13.92 ± 0.5	9.65 ± 0.1	9.67 ± 0.2	59.6	28.5
extractives (by difference)	11.52	11.45	6.83	35.5	24.3

a Cellulosic-lignin residue solid obtained after dilute acid pretreatment, under optimal conditions (85 mg H_2_SO_4_/g biomass, for 10 min at constant temperature of 150 °C), of deacetylated rice straw.

b Recovery of each component after dilute acid pretreatment.

c Recovery of each component after sequential process (deacetylation followed by dilute acid pretreatment).

As shown in Table 2, the cellulose fraction content in the pretreated solid (58.2% w/w) was 1.7 times higher than that found (34.7% w/w) in the raw biomass. When evaluating the recovery of components, a high CR (82%) was obtained after pretreatment with dilute acid. This value was only modestly reduced (69%) considering the entire sequential process (deacetylation followed by dilute acid pretreatment). As expected, lignin was the less affected component in terms of removal (8%) by the dilute acid pretreatment; however, it represented a low content (<20% w/w) in the pretreated solid due to the previous deacetylation. Reductions in acetyl groups, and ash content by 100, and 40%, respectively, were also observed when compared to the deacetylated rice straw. Thus, the pretreated solid composition in the present study can be beneficial for performing a high-solids enzymatic hydrolysis and subsequence 2G ethanol fermentation of C6 sugars due to the high cellulose and low interferences (hemicellulose, lignin, ash, and extractives) contents.

### 3.3 Effect of Gradual Feeding and VBM Reactor Utilization on High-Solids Enzymatic Hydrolysis

Gradual feeding of substrate, together with the use of a nonconventional reactor, enables efficient high-solids enzymatic hydrolysis, by improving mass transfer and mixing.^23^ A gradual feeding strategy (16 + 4 + 4% solids loadings) in a VBM reactor was established in our previous studies^12,22^ for an SSF process. In the present study, this strategy was used for the high-solids enzymatic hydrolysis of an SHF process using a pretreated solid (obtained by an optimized dilute acid pretreatment) as substrate. As demonstrated in Figure 3, the maximum concentrations of glucose (108 g/L), cellobiose (13 g/L), and xylose (8 g/L) were achieved after 48 h of hydrolysis, corresponding to a CCY of 78.6%. Both glucose concentration and CCY obtained in the present study are higher than those (92 g/L, 62%) previously achieved,^12^ which can be attributed to the more suitable composition and structure of the employed substrate (pretreated solid) for the enzymatic hydrolysis.

**Figure 3:**
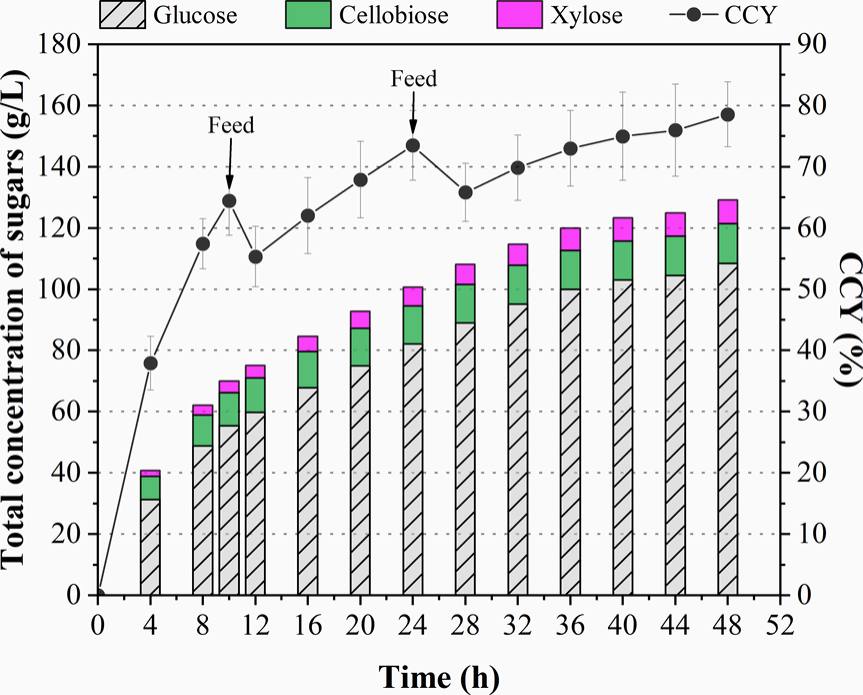
High-solids enzymatic hydrolysis (16 + 4 + 4% w/v) of pretreated solids of rice straw, in terms of total obtained sugars (glucose + cellobiose + xylose) and cellulose hydrolysis efficiency (CCY), in the vertical ball mill (VBM) reactor.

Additionally, the maximum values for the high-solids enzymatic hydrolysis in batch mode at solids loadings of 16% (75.7 g/L of glucose, CCY of 80%), 20% (85.9 g/L of glucose, CCY of 73%), and 24% (100.2 g/L of glucose, CCY of 72%) were obtained after 36 h of process. Thus, the glucose concentration in the enzymatic hydrolysate increased as the solid loading increased due to the higher available cellulose loading for the attack of enzymes. However, a decrease in the CCY was noted as the solids loading increased. This effect has been widely reported in literature, and its magnitude may vary depending on the biomass structure and composition, employed pretreatments, saccharification conditions, and reactor configuration.^10,34–37^ In general, this limited performance of the high-solids enzymatic hydrolysis in batch mode can be associated with difficulties in mixing (since a high-viscosity fibrous suspension is produced), mass transfer (due to low amount of free liquid water), product inhibition (by glucose and cellobiose), and higher cellulase adsorption.^18^ On the other hand, the gradual feeding strategy (16 + 4 + 4% w/v solids loading) in the VBM reactor improved the total fermentable sugar concentrations and CCY by 9 and 10%, respectively, as compared to the high-solids enzymatic hydrolysis at 24% w/v solids loadings in batch mode. Thus, this strategy in a nonconventional reactor enabled the production of a glucose-rich hydrolysate that can benefit fermentation performance in terms of ethanol concentration produced.

### 3.4 Effect of Nutrient Supplementation of Slurry/Hydrolysate Media on Ethanol Production in Shake Flasks

Figure 4 illustrates the fermentation profiles of the different evaluated media (slurry + nutrients, just slurry, hydrolysate + nutrients, and just hydrolysate) employing *K. marxianus* at 43 °C in shake flasks. Based on these profiles, the fermentative parameters were calculated and are summarized in Table 3.

**Figure 4:**
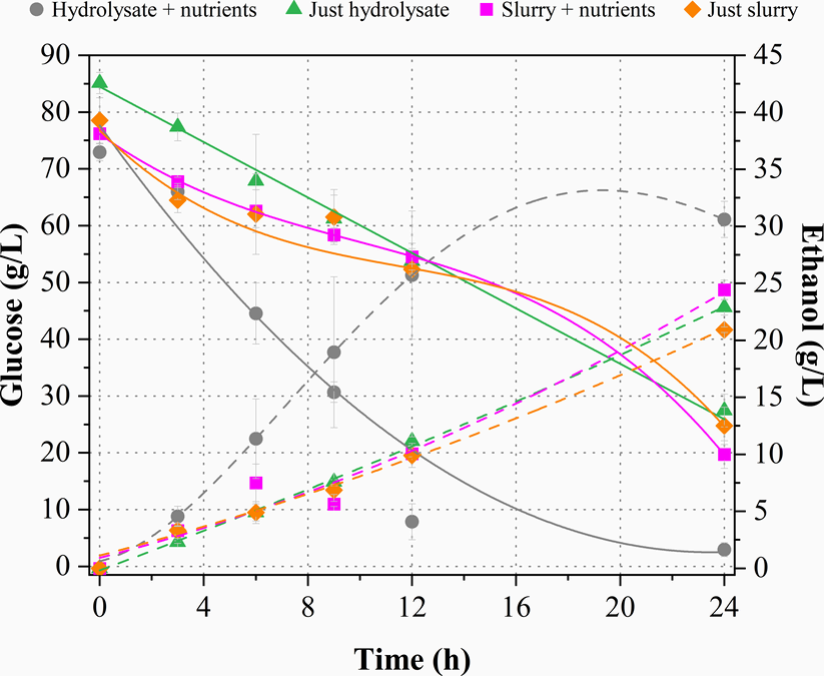
Kinetic profiles of glucose consumption (continuous lines) and ethanol production (dashed lines) by *K. marxianus* at 43 °C, evaluating nutrient supplementation and different fermentation media in shake flasks.

**Table 3:** Fermentative Parameters of Ethanol Production at High Temperature (43 °C), Employing *K. marxianus*

reactor type	shake flasks				vertical ball mill (VBM)	
fermentation medium	slurry		hydrolysate		semidefined medium	hydrolysate
nutrient supplementation	no	yes	no	yes	yes	yes
ethanol production (g/L)	21.0^b^	24.3^b^	23.0^b^	33.1^c^	28.6[Table-fn t3fn4]	36.6[Table-fn t3fn4]
glucose consumed (%)	67.9^b^	74.1^b^	69.3^b^	93.2^c^	80.0[Table-fn t3fn4]	97.1[Table-fn t3fn4]
$Y_{P/S}$ (g/g)	0.39^b^	0.43^b^	0.40^b^	0.46^c^	0.33[Table-fn t3fn4]	0.39[Table-fn t3fn4]
*Q*_P_ (g/L/h)	0.87^b^	1.02^b^	0.96^b^	1.74^c^	2.38[Table-fn t3fn4]	3.04[Table-fn t3fn4]
η (%)	76.3^b^	84.8^b^	78.4^b^	90.2^c^	64.7[Table-fn t3fn4]	76.5[Table-fn t3fn4]

a Value calculated considering highest ethanol pick: at 24 h.

b Value calculated considering highest ethanol pick: at 19 h.

c Value calculated considering highest ethanol pick: at 12 h.

Regarding glucose consumption profiles (Figure 4), it can be suggested that *K. marxianus* showed diauxic growth in the mediums containing slurry (regardless of nutrient supplementation), since an adaptive phase was observed between 9 and 16 h of fermentation. This time also coincided with a slight consumption of cellobiose (data not shown) that was present in the slurry (about 6 g/L), signifying a sequential consumption pattern of two carbon sources by *K. marxianus*. However, the residual solids in the slurry hindered the measurement of OD with the aim of verifying the cell growth profile. In the media containing hydrolysate (regardless of nutrient supplementation), glucose consumption practically presented a linear profile (Figure 4), emphasizing the suitability of this hydrolysate for fermentation by *K. marxianus*. In general, *K. marxianus* was able to assimilate glucose in both the hydrolysate and slurry, regardless of nutrient supplementation (Table 3). Particularly, almost complete glucose consumption (>90%) was observed in the hydrolysate + nutrients medium after 19 h of fermentation, while in the other evaluated media (just hydrolysate, just slurry, and slurry + nutrients), there was a residual glucose of approximately 30% even after 24 h. Therefore, the glucose consumption rate was favored by nutrient supplementation and the absence of solids from the slurry.

Regarding ethanol production (Figure 4), similar concentrations (about 23 g/L) were determined in the media containing just hydrolysate, just slurry, and slurry + nutrients. On the other hand, a higher ethanol concentration was obtained in the hydrolysate + nutrients medium, reaching a maximum peak of 33.0 g/L at 19 h, corresponding to a $Y_{P/S}$ of 0.46 g/g (Table 3). Thus, using the hydrolysate + nutrients medium resulted in a 74% improvement in ethanol production as compared to the other evaluated mediums, after 19 h of fermentation. Therefore, the lack of nutrients negatively affected the fermentation performance ( $Y_{P/S}$ , *Q*_P_, and η) of *K. marxianus*, which indicated an insufficient nutritional composition in both just hydrolysate and just slurry media, highlighting the need for nutrient supplementation to enhance the fermentation process.

In another work,^8^ the lack of nutrients had no significant impact on $Y_{P/S}$ , but rather *Q*_P_, when fermenting an enzymatic hydrolysate obtained with dilute acid-pretreated rice straw as substrate, emphasizing the role of biomass pretreatment in the whole process. Thus, pretreatment processes and conditions used in this study could vary the available nutrients in the slurry and the hydrolysate. Additionally, the presence of solids in the slurry further hindered the fermentation process, possibly by increasing the apparent viscosity of the medium and decreasing mass transfer.^38^ Hence, the challenges associated with using the slurry as a fermentation medium could be lessened by optimizing the operational conditions such as the agitation rate.

Based on the results obtained in shake flasks, the hydrolysate + nutrients medium was selected for performing the high-temperature fermentation in the VBM reactor, since it proved to support a highly (90%) efficient ethanol production, employing *K. marxianus*.

### 3.5 Effect of VBM Reactor Utilization on Ethanol Production

To evaluate the effect of the VBM reactor on the high-temperature fermentation of the hydrolysate + nutrients medium employing *K. marxianus*, a semidefined medium was used as a control under the same operational conditions. Figure 5 illustrates the resulting kinetic profiles of glucose uptake, cell growth, and ethanol, acetic acid, and glycerol production.

**Figure 5:**
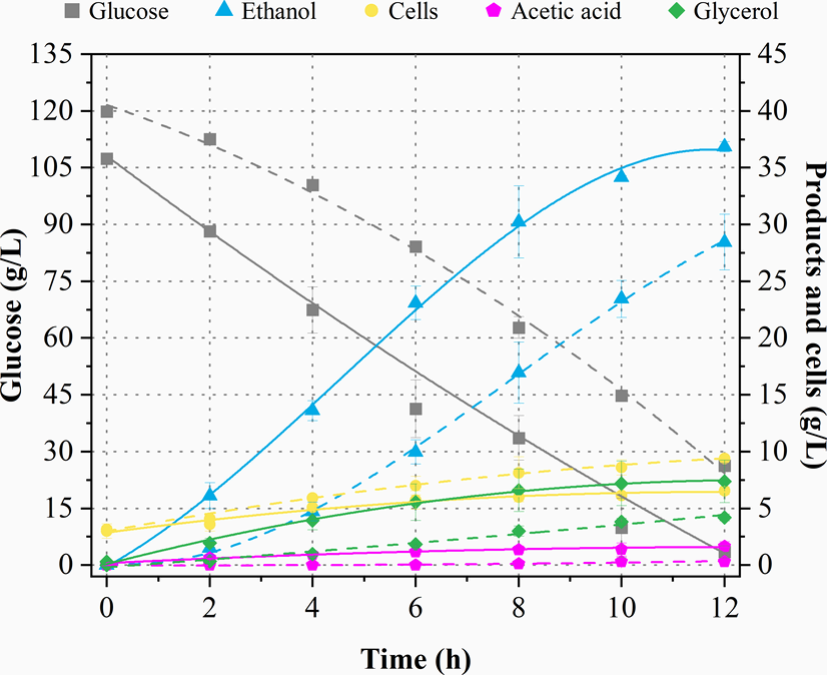
Fermentation profile of *K. marxianus* at 43 °C comparing fermentation media: hydrolysate + nutrients (continuous lines) and semidefined medium (dashed lines), both in the vertical ball mill (VBM) reactor.

In both the evaluated fermentation media (hydrolysate + nutrients and semidefined medium), the profile of glucose uptake was practically linear throughout the entire monitoring period (0–12 h) (Figure 5). Specifically, glucose uptake was almost total (97%) in the hydrolysate + nutrients medium, while 20% of the initial glucose concentration remained in the semidefined medium after 12 h (Table 3), indicating that the glucose consumption rate was higher in the hydrolysate + nutrients medium. This effect may be attributed to the presence of enzymes (cellulases) in the hydrolysate + nutrient medium, which can be considered as an additional nitrogen source, positively influencing the fermentation process. Moreover, it has been reported that the enzymatic extracts, Cellubrix and Novozyme 188, contained fermentation inhibitors (sorbitol and low molecular phenolic compounds), but these decreased to nontoxic levels due to dilution for hydrolysis and fermentation.^8^ Additionally, when comparing the glucose uptake in shake flasks and in the VBM reactor employing the hydrolysate + nutrients as fermentation medium, a slightly higher value was observed in the VBM reactor (Table 3). This indicated that the operational conditions used in the VBM reactor benefited the fermentative process in terms of substrate uptake.^22^ It is also worth mentioning that cells remained viable throughout the entire fermentation process.

In addition to glucose, the hydrolysate + nutrients medium also contained low amounts of cellobiose (12 g/L) and xylose (9 g/L), resulting from the high-solids enzymatic hydrolysis. Cellobiose was slightly (30%) consumed by *K. marxianus* after 12 h of fermentation (data not shown), since this thermotolerant yeast can metabolize various mono- and disaccharides, including glucose, fructose, sucrose, lactose, and galactose.^39^ However, a lack of xylose uptake was observed in the present study, probably because the uptake of this monosaccharide occurs following glucose depletion due to repression of the KmXYL1 gene for xylose reductase by glucose,^40^ therefore requiring more monitoring time to detect the decrease in xylose concentration.

Regarding ethanol production (Figure 5), it increased by 28% when employing the hydrolysate + nutrients medium (36.6 g/L) as compared to the semidefined medium (28.6 g/L) after 12 h of fermentation in the VBM reactor. As with ethanol titer, all fermentative parameters ( $Y_{P/S}$ , *Q*_P_, and η) obtained employing *K. marxianus* in the VBM reactor were higher when using the hydrolysate + nutrients as fermentation medium (Table 3). However, the obtained ethanol titer (36.6 g/L) is still <50 g/L, which is considered a reference value to make ethanol distillation technically and economically feasible.^41^ On the other hand, it is worth noting that *Q*_P_ reached a very high value (3.0 g/L/h) even when compared to *Q*_P_ (0.3–2.0 g/L/h) obtained also by fermentation of glucose-rich (∼110 g/L) hydrolysates employing a well-established yeast in the process, such as *S. cerevisiae*.^42,43^ These results suggest that the hydrolysate + nutrients medium exhibits promising characteristics for ethanol production by *K. marxianus* at high temperatures, with the process being enhanced in terms of *Q*_P_ using the VBM reactor, although further studies are required to optimize the operational conditions and consequently improve the process in terms of $Y_{P/S}$ and η. To our knowledge, the present study is the first time the VBM reactor was used for the SHF process, employing *K. marxianus*.

As can also be seen in Figure 5, two main byproducts (acetic acid and glycerol) were produced along with ethanol by *K. marxianus*. Acetic acid production achieved values of 1.67 and 0.32 g/L in the hydrolysate + nutrients and semidefined media, respectively. Glycerol production in the hydrolysate + nutrients medium (7.5 g/L) was also higher than that (4.4 g/L) found in the semidefined medium after 12 h of fermentation. Cells growth, on the other hand, was favored in the semidefined medium. These findings clearly show that the hydrolysate + nutrients medium provided a more suitable composition for directing the metabolic flux of *K. marxianus* toward ethanol production than the semidefined medium. In fact, for ethanol production under conditions of limited oxygen supply, *K. marxianus* accumulates acetic acid along with NADH when acetylCoA is needed (associated with low biomass production), triggering a cytoplasmic redox imbalance. Then, as a response to balance NADH oxidation, glycerol is formed.^44^ Since *K. marxianus* metabolism is sensitive to the oxygen consumption rate, it is possible to suggest that a metabolic control by adjusting the operational conditions of the VBM reactor (agitation or aeration) further orients the conversion of glucose into ethanol, enhancing $Y_{P/S}$ and η. Finally, the results obtained highlighted the importance of selecting the appropriate yeast for ethanol production at high temperatures (≥40 °C), which can be an alternative for reducing costs and operational difficulties at large-scale production.^13,14,45^

## 4 Conclusions

The dilute acid pretreatment conditions of deacetylated rice straw were successfully optimized, resulting in a pretreated solid with high cellulose content (58% w/w) and low hemicellulose and lignin contents. For the high-solids enzymatic hydrolysis of the pretreated solids in the VBM reactor, the gradual feeding strategy favored mixing and improved the CCY by 10% as compared to the control (high-solids enzymatic hydrolysis at 24% w/v solids content in batch mode), achieving a concentration of 129 g/L of total fermentable sugars. The fermentability of the glucose-rich hydrolysate at 43 °C was enhanced by supplementation with nutrients and the absence of residual solids in the medium. In the hydrolysate + nutrients medium, the higher $Y_{P/S}$ (0.46 g/g), *Q*_P_ (1.74 g/L/h), and η (90%) were achieved, employing *K. marxianus*. Finally, the use of the VBM reactor further increased *Q*_P_ (3.04 g/L/h) with an ethanol titer of 37 g/L, but more conditions need to be studied for improving $Y_{P/S}$ and η. Therefore, the defined sequential process and conditions of pretreatment, high-solids enzymatic hydrolysis, and high-temperature fermentation were very promising and could contribute to the sustainability of 2G ethanol production at a large scale.

## Supplementary Material



## Notes

Effects of different conditions of dilute acid pretreatment on the composition of deacetylated rice straw and hemicellulosic hydrolysate; response surfaces described by the adjusted mathematical models representing CR, MR, HHE, *C*_Fur_, and *C*_Phe_; and desirability response surface (PDF)
