# Supplementary material for: Efficient 2G Ethanol Production via Optimized Dilute Acid Pretreatment, High-Solids Enzymatic Hydrolysis, and High-Temperature Fermentation
Source: ACS Omega. 2026 Jan 8;11(2):3518–28. doi: 10.1021/acsomega.5c11192 (PMC12824952; doi:10.1021/acsomega.5c11192)
Supplement: Supplementary file 1 [file ao5c11192_si_001.pdf]

Supporting Information

Efficient 2G Ethanol Production via Optimized Dilute Acid Pretreatment, High-Solids Enzymatic Hydrolysis, and High-Temperature Fermentation

Isabela S. Ferreira [a]; Miguel A. D. Flores-Alarcón [a]; Inês C. Roberto [a,*]

[a] Department of Biotechnology, Engineering College of Lorena, University of São Paulo (USP), Estrada Municipal do Campinho, N° 100, Campinho, Lorena, SP 12602-810, Brazil.

*Corresponding author email address: iroberto@usp.br

Table S1. Effects of different conditions of dilute acid pretreatment on the composition of rice straw and hemicellulosic hydrolysate, according to a face-centered central composite design (FC-CCD).

Assay[a]	Independent variables[b]		CSF[c]	Composition																				
				Pretreated solid (g/100 g)					Hemicellulosic hydrolysate															
									Sugars (g/L)			Acetic acid (g/L)	Furans (mg/L)		Phenolics (mg/L)									
	X_1	X_2		Glucan	Hemi	Acetyl	Lignin	Ash	Glucose	Xylose	Arabinose		Furfural	HMF	Furoic acid	Gallic acid	Vanillyl alcohol	Pyrocatechol	Vanillic acid	Syringic acid	Vanillin	Syringaldehyde	p-Coumaric acid	Ferulic acid
1	150	0.5	1.44	60.55	9.84	0.00	16.49	8.09	1.47	14.63	4.41	0.16	1.19	15.38	178.53	0.00	53.52	1090.17	9.20	0.39	0.00	8.02	5.36	8.01
2	170	0.5	2.03	63.36	4.19	0.00	19.31	9.39	3.01	13.89	3.32	0.42	2.55	86.90	392.03	0.00	91.43	5979.40	16.90	0.00	2.95	8.91	5.19	2.35
2 (Rep)	170	0.5	2.03	64.18	3.92	0.00	19.13	9.44	2.77	13.49	3.17	0.30	2.89	89.41	376.23	0.00	102.24	3538.30	17.73	0.00	0.00	4.61	6.37	2.89
3	150	1	1.67	64.02	3.71	0.00	18.89	9.16	3.43	17.76	4.06	0.29	1.67	31.77	233.50	0.00	73.67	2509.61	11.52	0.00	2.73	0.00	2.11	2.97
3 (Rep)	150	1	1.67	65.52	5.46	0.00	18.25	8.73	2.95	18.10	4.37	0.31	1.20	20.44	288.08	0.00	57.25	933.24	9.20	0.00	1.86	3.98	2.29	5.10
4	170	1	2.26	69.34	1.90	0.00	22.19	3.68	5.85	9.53	2.53	0.00	0.00	127.85	293.58	0.00	92.62	4960.48	28.94	1.94	2.92	0.00	2.94	1.16
5	150	0.75	1.60	63.41	5.89	0.00	18.48	9.09	3.02	17.57	4.22	0.23	1.26	24.52	176.53	0.00	59.61	1902.96	8.96	0.00	0.00	4.83	3.30	6.03
6	170	0.75	2.19	65.44	2.50	0.00	19.73	8.66	3.86	12.38	3.12	0.39	3.14	90.71	281.13	0.00	104.89	4933.65	21.39	0.00	0.00	9.01	4.08	2.03
7	160	0.5	1.74	62.83	6.46	0.00	18.13	8.63	2.25	15.94	3.99	0.00	2.26	39.88	215.90	0.00	82.68	1603.58	11.89	0.00	0.00	7.67	7.20	5.85
8	160	1	1.97	64.53	2.93	0.00	19.28	9.42	3.97	17.25	3.95	0.25	2.46	50.76	260.48	0.00	100.34	2443.76	17.04	0.00	1.99	9.09	2.32	2.68
9 (Cp)	160	0.75	1.90	67.30	3.99	0.00	18.68	7.56	3.24	15.59	3.50	0.00	2.66	61.81	322.42	0.00	85.76	3333.81	16.12	0.00	0.00	7.24	3.69	3.01
10 (Cp)	160	0.75	1.90	65.89	4.45	0.00	19.01	6.38	3.16	16.96	3.92	0.27	2.20	41.70	271.90	0.00	79.27	1802.87	14.92	0.00	0.00	7.21	3.46	4.78

[a] Rep = replicate, Cp = central point. [b] X_1 = temperature (°C), X_2 = H_2SO_4 concentration (% m/v). [c] CSF = combined severity factor.

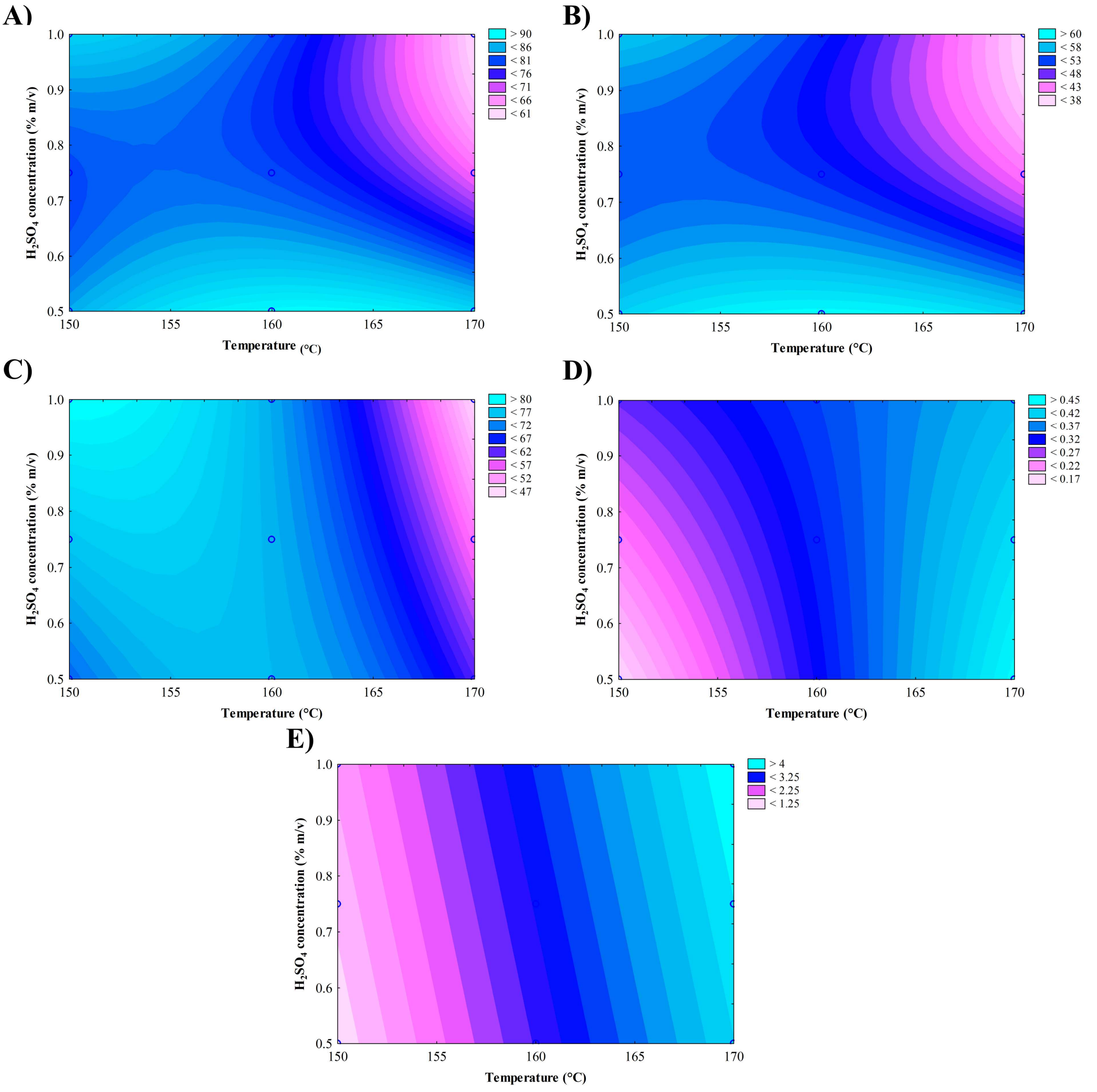

Figure S1. Response surfaces described by the adjusted mathematical models representing: A) cellulose recovery, B) mass recovery, C) hemicellulose hydrolysis efficiency, D) concentration of furans, and E) phenolics, as a function of temperature (X_1), and H_2SO_4 concentration (X_2) from dilute acid pretreatment in the 0.5-L stainless steel reactors.

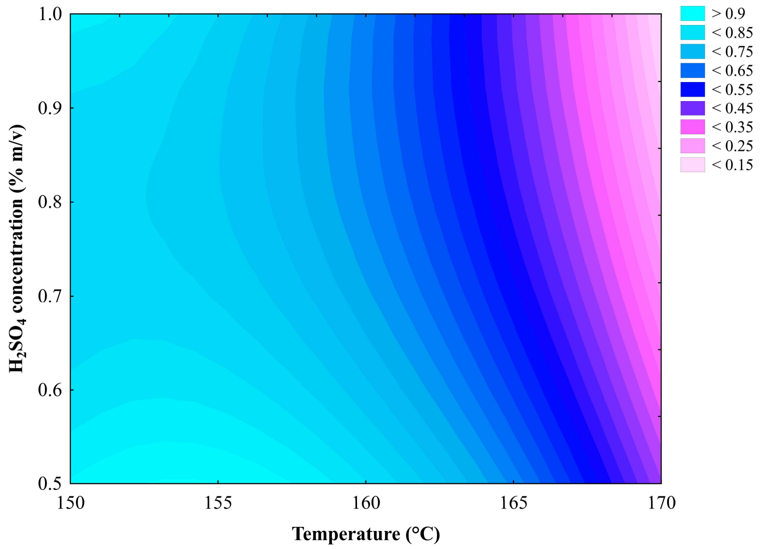

Figure S2. Desirability response surface obtained by maximizing the cellulose recovery, mass recovery, and hemicellulose hydrolysis efficiency (%) while minimizing the concentration of furans and phenolics (g/L).
